# Capturing the complexity of sex differences requires multidimensional behavioral models

**DOI:** 10.1038/s41386-019-0424-6

**Published:** 2019-06-04

**Authors:** Cody A. Siciliano

**Affiliations:** 1 The Picower Institute for Learning and Memory, Department of Brain and Cognitive Sciences, Massachusetts Institute of Technology, Cambridge, MA 02139 USA; 2 Department of Pharmacology, Vanderbilt University School of Medicine, Nashville, TN 37232 USA

**Keywords:** Reward, Motivation

Over the last decade we have seen a sharp rise in opioid abuse, addiction, and overdose deaths in the United States [1]. As this epidemic continues to worsen, the need for additional effective pharmacotherapeutics to complement opioid maintenance therapy becomes increasingly important. One of the major focuses of preclinical addiction work has been to identify and develop therapeutic drugs to treat substance use disorders; however, most of this work has been conducted in male subjects, potentially neglecting issues specific to female subjects. For example, opioids can have differential effects between sexes with men showing higher rates of opioid abuse [2], whereas women experience more of the negative side effects associated with opioid use [3]. While it is tempting to conclude from the epidemiological data that men are more vulnerable to opioid abuse, it is important to understand that sex is not a behavioral determinant on its own. Rather, sex interacts with environmental conditions and stimuli to produce sex-specific effects in certain contexts. Understanding the neurobiology of sex differences will require holistic behavioral models that capture the complexity of sex by environment interactions underlying sex-specific behaviors.

While preclinical studies have reported mixed results regarding sex differences in opioid self-administration and relapse [4, 5], the prevailing hypothesis is that female rodents take more opioids than males [6], which conflicts with the clinical literature where men show higher rates of opioid abuse [2]. In this issue of *Neuropsychopharmacology*, Townsend and colleagues [7] explore this discrepancy by examining the reinforcing effects of the potent synthetic opioid fentanyl and the palatable liquid food Ensure in male and female rats across several schedules of reinforcement. Using an innovative combination of behavioral assays, they show that there are indeed striking sex differences in opioid self-administration—however, the directionality of these effects depends on the schedule of reinforcement under which fentanyl is presented. Under a fixed-ratio five schedule of reinforcement, fentanyl maintained higher rates of responding in  female rats as compared to males. Similarly, females showed greater motivation to self-administer both fentanyl and Ensure during a behavioral economics task where the ratio requirement to obtain fentanyl was increased over days. Despite the fact that females took more fentanyl in both of these experiments, when rats were presented with the choice between fentanyl and Ensure males chose fentanyl more frequently than females, demonstrating that the context within which drugs are presented—especially in regards to alternative reinforcers—can alter the way the rats allocate their effort. Valuing drugs over natural reinforcers is a defining characteristic of addiction, and one that is often overlooked in animal studies where, in many cases, access to drugs is given in isolation from other reinforcers.

These findings highlight the potential pitfalls of making general conclusions based on a single behavioral procedure and demonstrate the critical need to study drug effects across multiple schedules of reinforcement and environmental conditions. Indeed, had the authors only examined sex differences in self-administration when a single reinforcer was available, or only examined choice behavior, they would have come to different, and incomplete, conclusions. The use of multidimensional behavioral analyses is especially important when establishing sex-specific factors that contribute to drug consumption, appetitive responding, and motivation, all of which are controlled by different neural circuits and can change independently of one another. Here, Townsend et al. find that instead of generalized effects of sex on responding for fentanyl, sex effects are specific to the schedule under which responding for fentanyl is reinforced, and the context that it is presented. Further, as mentioned above, this work highlights a flaw in many addiction studies: the availability of alternative reinforcers can give important information about how laboratory animals and humans value drug rewards and may be a key factor in testing putative pharmacotherapies for addiction. Together, one of the most important factors illuminated by Townsend et al. [7] is that the level of drug consumption does not predict the relative value of the drug when self-administration is presented as a choice between two reinforcers. Finally, the findings that male rats show greater preference for fentanyl than females under choice conditions is consistent with higher rates of opioid abuse in men suggesting that studies that allow laboratory animals to choose between drug and natural reinforcers may have greater predictive validity of abuse rates in humans.

The dissociation between sex differences in drug consumption and choice echoes a founding principle of the behavioral pharmacology field which is often overlooked in behavioral neuroscience—that natural and drug stimuli do not have universal behavioral actions, but instead are dependent on the schedule under which the stimuli are presented [8, 9]. Importantly, this study extends these principles to sex differences, and gives an empirical demonstration that sex differences in opioid reinforcement do not manifest themselves as generalized effects. Rather, there are complex interactions between sex and environmental factors that in some cases can drive differential behaviors between the sexes. Keeping in mind that the behavioral effects of stimuli are dependent on schedule, and that sex is not a behavioral determinant, but rather a variable that interacts with a wide array of environmental factors, will aid in the design of studies examining sex differences in behavioral strategies, as well as their interpretation.

Moving forward it will be critical to not only increase the prevalence of studies including female subjects, but to also understand the breadth of the sex differences and the female-specific factors that underlie opioid abuse. Importantly, it may be more relevant to ask how sex and environment interact, rather than to search for universal behavioral differences between sexes.

## Funding and disclosure

C.A.S. is supported NIH grant K99 DA04510 (NIDA), and a NARSAD Young Investigator Award (Brain and Behavior Research Foundation).
